# Intussusception in the Geriatric Population: A Case Report

**DOI:** 10.7759/cureus.24663

**Published:** 2022-05-02

**Authors:** Benoit Boucher, Orlando Fleites, Rio Varghese, Julius Myuran Nagaratnam, Fabrice Yabit, Juaquito Jorge

**Affiliations:** 1 Surgery, Saint James School of Medicine, Chicago, USA; 2 Surgery, Saint James School of Medicine, Park Ridge, USA; 3 Surgery, Saint James School of Medicine, West Virginia, USA; 4 Surgery, Avalon University School of Medicine, Chicago, USA; 5 General and Bariatric Surgery, West Suburban Hospital, Oak Park, USA

**Keywords:** geriatric population, transition point, adenoma, exploratory laparotomy, small bowel obstruction, intussusception

## Abstract

Recurrent abdominal pain in the adult population is a complex symptom with a broad spectrum of diagnoses. The diagnosis of intussusception in the elderly is considerably rarer than in the younger population. High clinical suspicion is required, and imaging is needed for confirmation. Here, we present and discuss the clinical course and management of an 82-year-old female who underwent small bowel resection following recurrent intussusception that was confirmed by imaging and at the time of surgery. The patient was known for having a history of polyps, and the pathology report described a large tubulovillous adenoma found on the resected small bowel specimen. The patient was discharged after surgery with complete remission. This case report intends to explore the importance of surgical intervention versus conservative management in a patient with a similar clinic presentation. This report also intends to highlight the importance of surgical intervention to prevent intussusception-related complications and reduce patient mortality further.

## Introduction

Intussusception is the invagination of a proximal portion of the intestine into its distal aspect [1], often referred to as telescoping [2]. The condition is typical among pediatrics and infants, with a peak incidence occurring in infants of 5-7 months of age with an incidence of 74 per 100,000 [3,4] in children under one year old. Typical clinical presentations include colicky abdominal pain, mucus or blood-tinged stool, emesis, diarrhea [5,6], and a palpable mass on the abdomen during physical examination [6]. Small bowel intussusception is more expected in the pediatric population and is rare among adults, accounting for less than 5% of all cases [7].

The causes of intussusception in adults are diverse. Many etiologies such as neoplasms, enteric autoimmune pathologies, history of intra-abdominal surgeries, and gynecologic conditions such as endometriosis are known to cause intussusception [7]. The common complications associated with this condition include small bowel obstruction (SBO), bowel ischemia, necrosis, bowel perforation with peritonitis, and sepsis [8], as a result requiring urgent care [1,9].

Management for adult patients with presenting signs and symptoms suggestive of intussusception or other obstructive bowel pathologies often involves detection via an abdominal computed tomography (CT) scan [10,11], which is often the gold standard for early detection [7,11]. In contrast to the pediatric population, where endoscopy is diagnostic and usually therapeutic, intussusceptions are beyond the reach of endoscopes in their geriatric counterparts. The preferred surgical intervention is an exploratory laparotomy with bowel resection and anastomosis or laparoscopic bowel resection with bowel anastomosis [12]. Surgical intervention is required due to possible bowel obstruction, ischemia, or necrosis [13].

## Case presentation

An 82-year-old female presented to the emergency room complaining of cloudy, odorous urine, weak stream, and generalized weakness for two days. She also reported constipation for approximately one month and poorly tolerating oral intake due to nausea and vomiting. She denied abdominal pain, hematochezia, melena, excessive belching, bloating, diarrhea, hematuria, and dysuria.

The patient's past medical history is significant for recurrent UTI, well-controlled DM2, atrial fibrillation, multiple CVA with left-sided residual impaired mobility of lower and upper extremities, and hypertension. Surgical history is notable for hysterectomy, oophorectomy, appendectomy, and hernia repair. The patient denied illicit drug use, along with the usage of alcohol. Medication regimen include atorvastatin, Eliquis, amiodarone, enalapril, hydrochlorothiazide, insulin glargine, senna, bisacodyl, pantoprazole, labetalol, metoprolol, and warfarin. Allergies were notable for codeine, penicillin, quinine, and contrast media.

CT scan revealed that a long jejunal segment was intussuscepted, with stool impaction in the colon. Although the patient denied a family history of gastrointestinal malignancy, concerns were raised upon the patient mentioning that polyps were found 10 years ago on prior colonoscopy. The patient could not recall what type or where were those polyps located. The patient asked for conservative management, but an air enema was not a viable option and is contraindicated for this patient, given the proximal nature of the intussusception and age. The gastroenterology specialist started the patient on saline enemas, and the patient was educated on how to use saline enemas for fecal evacuation regularly once discharged. The patient was ultimately discharged upon imaging, indicating improvement of both stool impaction and intussusception since starting the saline enemas. 

The patient presented to the emergency department five months later complaining of vomiting, nausea, lack of solid feces with enemas, and purulent urination that started three days before her visit. Laboratory results revealed elevated liver enzymes and leukocytosis, and urinalysis was positive for nitrites, leukocytosis, red blood cells, and bacteria. The patient denied any recent diet changes or travels and consistently used her enemas but noted that her stools are now watery. The patient refused surgical evaluation and was discharged on Bactrim to treat acute cystitis and Zofran to control nausea and vomiting.

**Figure 1:**
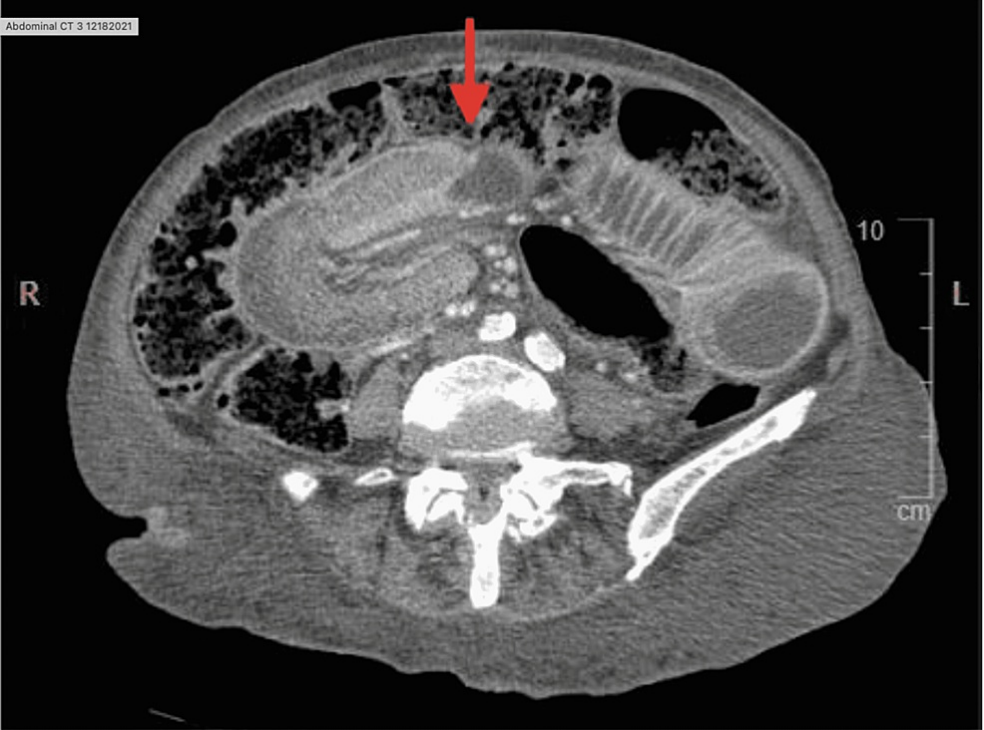
CT with intravenous contrast of the abdomen showing the transition point Red arrow: transition point

The patient returned to the emergency room in acute distress three months later, complaining of generalized abdominal pain, constipation, nausea, and vomiting for a month with a physical examination revealing mild abdominal distension and disclosed still being compliant with her enemas. Laboratory results were normal. CT with IV contrast (Figure 1) revealed small bowel obstruction secondary to intussusception in the right abdomen distal to the dilated bowel loops (up to 4.6 cm in distension), anasarca, distended urinary bladder, and pericholecystic fluid with mild wall thickening and a 3.3 cm cyst in the liver. The patient agreed to surgical intervention and was scheduled for an exploratory laparotomy. The risks and benefits of the procedure were discussed with the patient, and consent was obtained.

**Figure 2:**
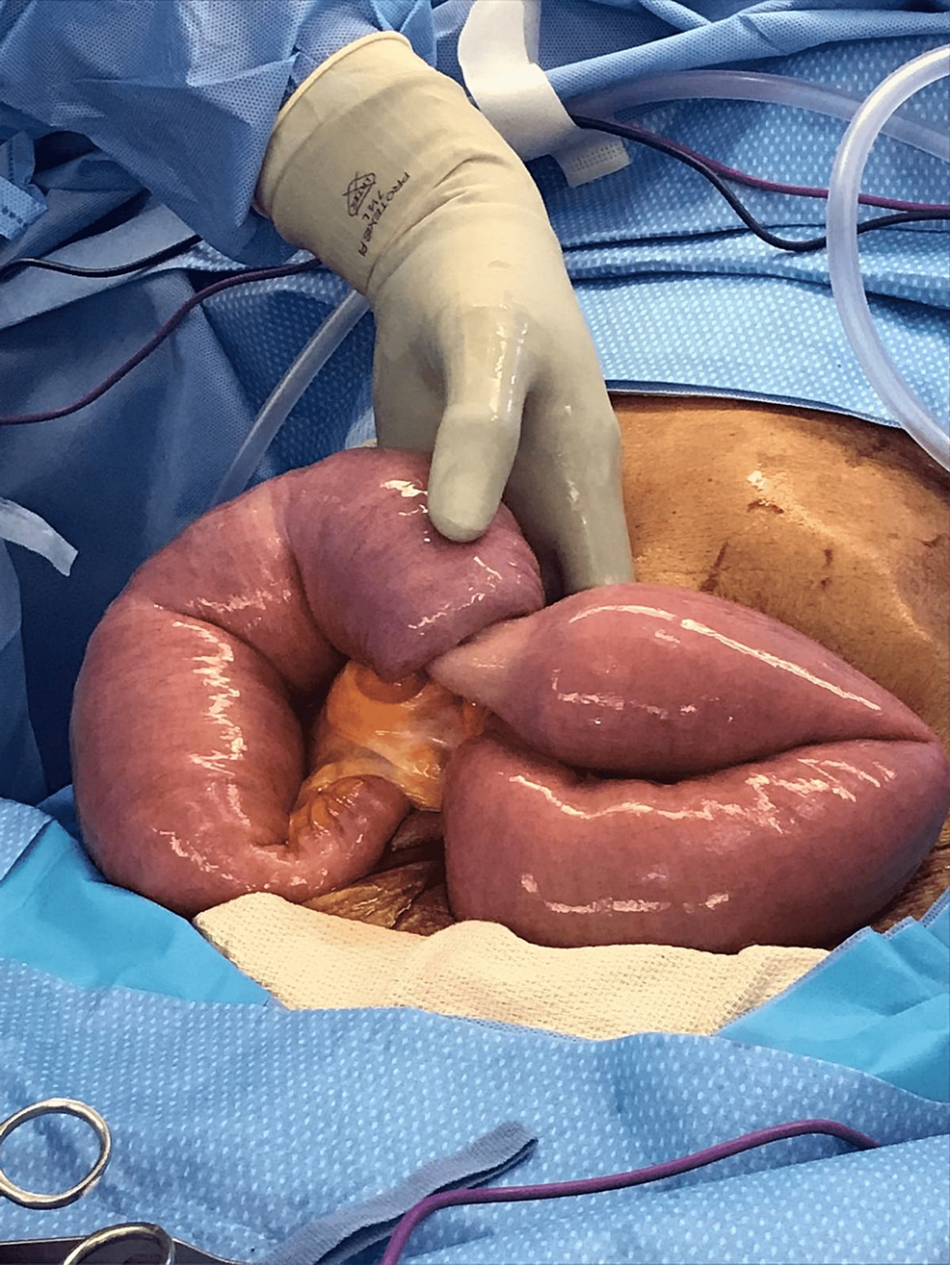
Small bowel transition point

Exploratory laparotomy was performed under general anesthesia with small bowel resection and primary anastomosis. Dilation of the stomach via insufflation was performed, the proximal small bowel was noted, the small bowel was eviscerated, and the transition point (Figure 2) was exposed. The telescoped portions of the bowel were resected, and anastomosis was created. The resected specimen was sent to pathology for further analysis, and the rest of the small bowel running from the ligament of Treitz down to the ileocecal valve was examined and showed no abnormalities. No adhesion, signs of bowel perforation or ischemia, or any other complications were noted intraoperatively, and the patient was maintained on a small bowel rest regimen, DVT prophylaxis, and antibiotics for 24 hours postoperatively. Bowel functions started convalescing on postoperative day 2. The pathology report returned positive for tubulovillous adenoma in the jejunum measuring 6 × 5.3 × 2.2 cm without evidence of cancer and negative dysplasia in all 12 regional lymph nodes analyzed. The margins of the specimen were free of dysplasia. Upon normal serial abdominal X-ray and return of bowel functions, the patient was discharged.

## Discussion

Intussusception is the overlapping of a proximal segment of the bowel into the lumen of its distal segment [14]. Adult intussusception cases typically have a malignant etiology, from which the abnormal growth serves as the lead point. At the transition point, peristaltic contractions constrict and relax the lumen, allowing for the invagination of the proximal segment into the distal bowel [2]. Adult intussusceptions account for less than 5% of all cases [7], from which 52% localize in the small bowel [15]. Intussusception caused by detectable structural lesions such as adhesions, inflammatory bowel diseases, Meckel's diverticulum, or neoplasm is called secondary intussusception [13]. The classic presentation of intussusception involves abdominal pain, currant jelly stools, and palpable tender mass, which is not generally seen in adult intussusception. Repeated bouts of nonspecific intermittent abdominal pain are a common complaint in adult patients diagnosed with intussusception [16].

The cause of our patient's adult intussusception was a large tubulovillous adenoma, measuring 6 × 5.3 × 2.2 cm, with no evidence of malignancy. Tubulovillous adenomas have a combination of tubular and villous makeup, with the latter alone having the more eminent propensity for malignancy [17]. Tubulovillous adenomas are poorly differentiated tumors and benign. The early stages of the adenoma are asymptotic, but unexpected growth can cause morbidity and ultimately become malignant. Treatment guidelines for tubulovillous adenomas require an extensive bowel resection to prevent the neoplasm from developing into adenocarcinoma. SBO due to a tubulovillous adenoma in the jejunum accounts for less than 2% [18], while 70% are secondary to adhesions from previous abdominal surgeries. Abdominal pain is considered the most common symptom for a patient with SBO, generally due to either partial or complete obstruction. Partial SBO will still have flatus and some stool traveling through the intestine. In contrast, complete SBO will have bowel obstination with an empty rectum [19].

Abdominal computed tomography (CT) scan is the gold standard for diagnosing intussusception and SBO. It is the most sensitive imaging of choice due to its ability to distinguish the absence or presence of a lead point in intussusception. Radiographic images of a "target sign," "bull's eye," or "doughnut sign" are auxiliary in diagnosing intussusception. A diagnostic CT scan for SBO will show dilated bowel loops with a transition point [20]. CT scan can be a reliable indicator of intussusception and SBO, although determining the underlying etiology can remain unclear through imaging.

## Conclusions

Although rare in the geriatric community, small bowel intussusception in the majority of cases results from a pathological lead point. Such was the case for our 82-year-old patient, who was found to have a sizeable tubulovillous adenoma albeit devout of any malignancy. This case displayed the importance of advocating surgical over conservative management since nine months of enemas did not initially solve the condition. Air enema remained an unviable option throughout the course in adult and geriatric populations due to the proximity of the intussusception that air could not reach. Exploratory laparotomy remains the curative option of choice in any nonpediatric population and pathology to be at the frontline for possible further treatment based on the etiology report of the lead point.
